# Esterification process catalyzed by ZSM-5 zeolite synthesized via modified hydrothermal method

**DOI:** 10.1016/j.mex.2018.03.004

**Published:** 2018-03-23

**Authors:** Omar Ben Mya, Mohammed Bita, Ilyas Louafi, Assia Djouadi

**Affiliations:** a Process Engineering & Petroleum Chemistry Department, University of El Oued, 39000, El Oued, Algeria; b Valorisation and Technologies of Saharan Ressources (VTSR) Laboratory, University of El Oued, 39000, El Oued, Algeria

**Keywords:** ZSM-5 catalyst, Oleic free fatty acid (OFFA), Esterification, Biodiesel, Sulfuric acid, Oleic acid methyl ester (OAME)

## Abstract

A modified hydrothermal method for ZSM-5 synthesis was described. The crystals gave a typical pattern (2θ at around 22.5°, 24.0° and 29.8° corresponding to the major peaks of (501, 303 and 503 crystal surfaces)), which indicated that the subnanocrystals could have the primary structure of MFI-type zeolites. the FT-IR spectra of subnanocrystals which have the primary structure of MFI zeolites. Oleic acid methyl ester (OAME) was prepared via a rapid derivatization procedure. the acidic strength is determined by the zeolite crystal structure and the higher esterification rate of ZSM-5 can be attributed to its stronger acidity compared to H_2_SO_4_, especially after 50 min of reaction. ZSM-5 can be an excellent substitute to sulfuric acid which caused corrosion and equipments damage.

• Zeolites are popular industrial catalysts comprised of crystalline microporous materials.
• A modified hydrothermal method was used for ZSM-5 synthesis.
• Synthesized ZSM-5 has a typical subnanocrystals structure corresponds to MFI- type Zeolite.
• ZSM-5 can be an excellent substitute to sulfuric acid for the catalysis of esterification reactions.

## Graphical abstract

**Figure:**
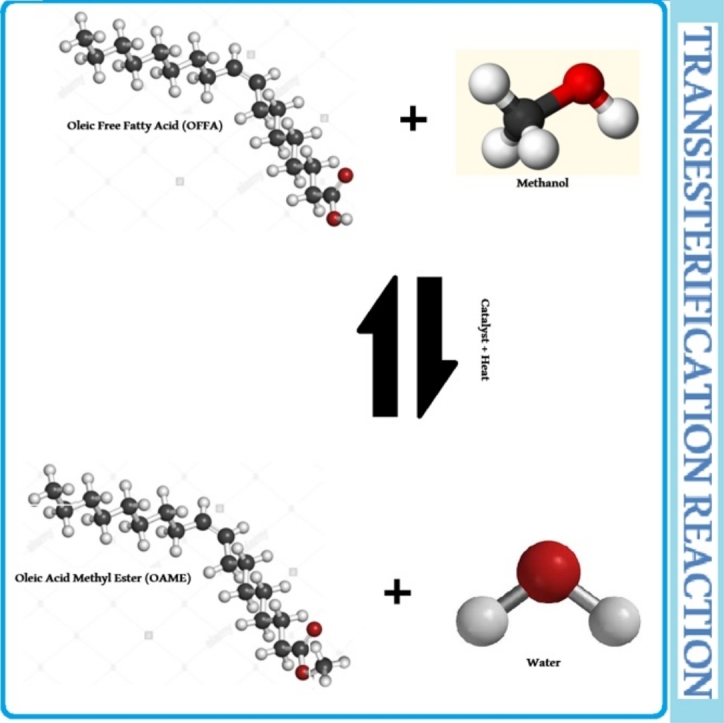

## Introduction

Zeolites are popular industrial catalysts comprised of crystalline microporous materials of different molecular sizes and shapes. They are widely used in oil refining, petrochemical production and organic synthesis of important chemicals. The intricate channel structure of zeolites provides selectivity based upon shape and polarity, and is particularly effective for reactant molecules with a kinetic diameter below 10 A° [1]. ZSM-5 is a representative of a new class of high-siliceous zeolites which exhibit exceptional catalytic properties and performance in oil refining process [[2], [3]]. Biodiesel is an oxygenated diesel fuel made from vegetable oils and animal fats by conversion of free fatty acids (FFA) to esters via transesterification [4], it was an alternative to fossil fuels, having negative environmental consequences and concerns about petroleum supplies, net energy gain, have environmental benefits, be economically competitive, and be producible in large quantities without reducing food supplies [5]. This paper explored the potential of ZSM-5 catalysts in converting of low value, short-chain oleic free fatty acid (OFFA) to oleic acid methyl ester (OAME) biodiesel.

## Experimental

### Synthesis and characterization of ZSM-5 catalysts

A modified hydrothermal method for ZSM-5 synthesis can be described as the following: Aqueous amount of Al_2_O_3_ (0.0018 mol), 0.075 mol of NaOH, Aqueous amount of 0.030 mol H_2_SO_4_ (96%), 4 g of (Ethane-1,2-diyldinitrilo) tetraacetic acid (EDTA) and 25 ml of SiO_2_ 30 wt.% (0.15 mol) were mixed and stirred for 30 min, pH should be in the 11–12 range until formation of zeolite gel. The filtered gel was placed in an oven at ∼95 °C for a week. After a week of heating, the product was gravity filtered and rinsed with water until the pH is about 8 and dried in air. To eliminate organic compounds and zeolite formation, the powder was calcined under 800 °C for 2 h at a rate of 10 °C/min. X-ray powder diffraction spectrum was obtained using D8 Advance Bruker X-ray diffractometer by scanning from 10 to 50°. FT IR spectra were obtained using a SHIMADZU 8400 s spectrometer in the range of 400–4000 cm^−1^.

### Esterification of oleic free fatty acid [6]

OAME was prepared via a rapid derivatization procedure. An aliquot (35.15 ml) of OFFA, 20 ml of methanol and 1.77 ml sulfuric acid solution (10 Wt%) were added into the Pyrex screw-cap tube (16 × 125 mm). The tube was vortexed for 10 s before being stirred to become homogeneous under 85 °C water bath for 15 min for acidic esterification. Afterwards, catalysis tests were carried out in the reactions and performed in a round-bottom glass flask with two-neck vol–250 ml equipped with a reflux condenser in a crystallizer. An excess of methanol was added to the flask containing the oleic acid in order to shift the equilibrium towards ester formation with a molar ratio of 1:4.5 (oleic acid:methanol). 4.5 (oleic acid:methanol). ZSM-5 catalyst (10 wt.% by weight, based on the oleic acid) was added to catalyze the esterification without it depends on the concentration of the reaction substrate. The crystallizer is placed on a hot plate about 70 °C with magnetic stirring about 450 rpm, maintaining the stability of the degree of the temperature through the thermocouple installed. Connecting the condenser in a small submersible pump placed in a water bath containing an amount of ice. This system condenses the vapor from the reaction to maintain the amount of the reactants. The esterification reaction is started and stay for three hours, several samples are taken at different times (0:10:30; 30:30:180 min) by Pasteur pipette, the samples are then separated by centrifuge. In order to regularize biodiesel acidity, OAME was placed in a separator funnel 60 ml, washed with distilled water and then with dilute solution of sodium bicarbonate, until having a value (PH = 7). The tube containing the OAME extract was centrifuged at 1500*g* for 2 min and 1 μl of hexane phase was injected for GC analysis. Results are obtained from GC data and the conversion of OAME was calculated by the formula [7]:

(1) $$\text{OAME conversion}\%=\frac{\text{OAME after conversion}\;(mol)}{\text{Oleic acid}\;\text{in}\;feedstock\;(mol)}\times 100\%$$

## Results and discussion

### ZSM-5 zeolite characterization

**Fig. 1:**
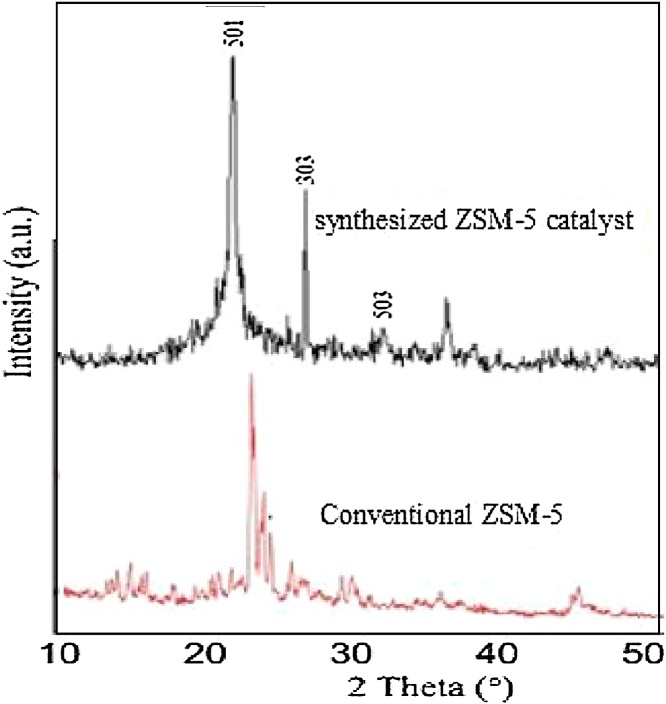
Comparison between Conventional ZSM-5 [8] and synthesized ZSM-5 catalyst XRD patterns.

Fig. 1 shows conventional(standard) and synthesized ZSM-5 diffractograms. The crystals gave a typical pattern, this fact can be seen from 2θ at around 22.5°, 24.0° and 29.8° corresponding to the major peaks of (501,303 and 503 crystal surfaces), which indicated that the sub nanocrystals could have the primary structure of MFI-type zeolites [[9], [10]]. synthesized ZSM-5 has a high intensity peak at 22.5° compared to commercial ZSM-5. Furthermore, synthesized ZSM-5 also has other peaks. This is because the degree of crystallinity of synthesized ZSM-5 is most important than conventional one [11].

Crystallization temperature of calcination is one parameter that plays an important role on the properties of the catalyst. The good crystallinity of the ZSM-5 catalyst proves that the choice of the calcination temperature of 800 °C for 2 h is just [10].

**Fig. 2:**
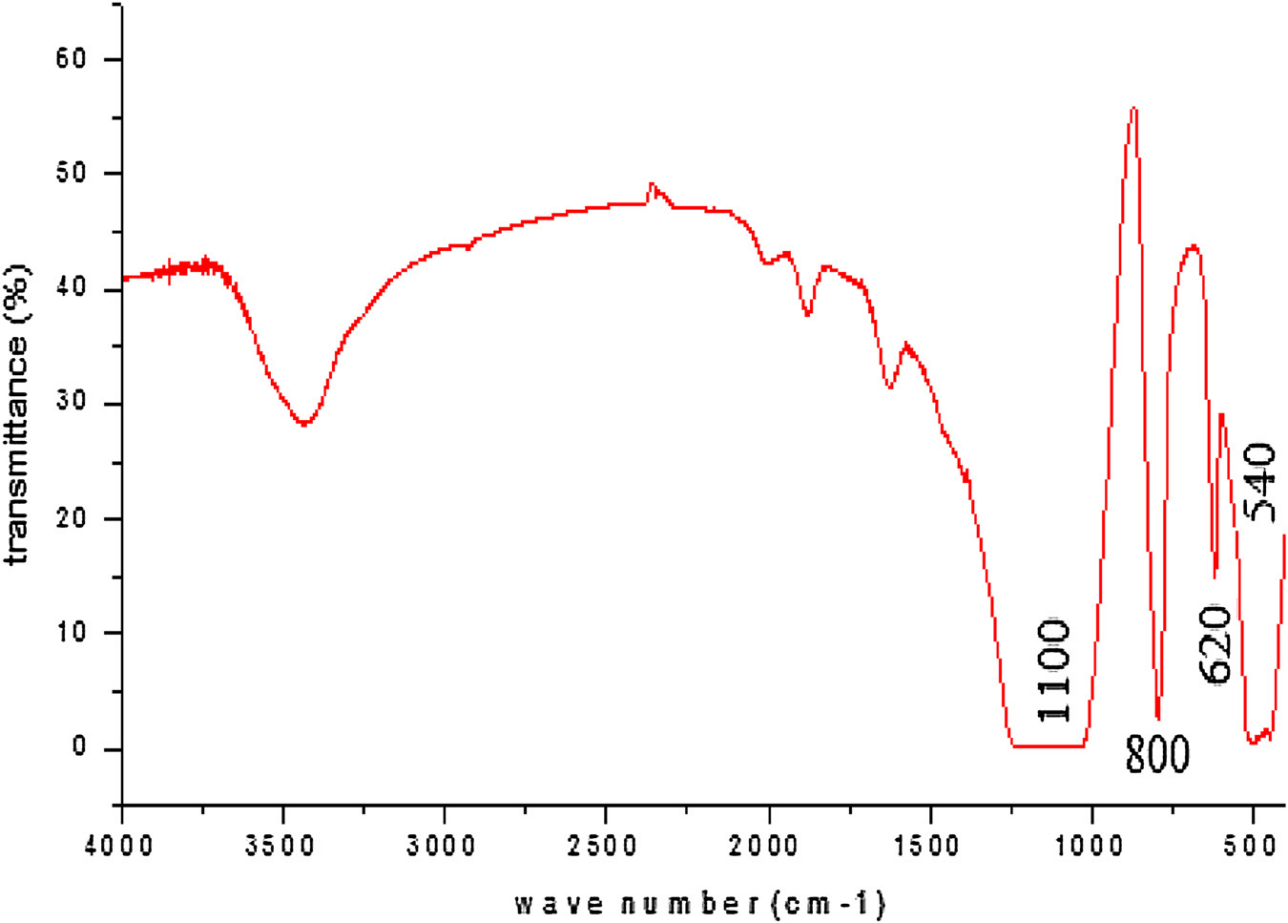
FT-IR spectrum of synthesized ZSM-5.

FT-IR spectra of zeolite lattice vibration modes are depicted in Fig. 2 in the range at 1100 cm^−1^ and 540 cm^−1^, all spectra of the samples show a typical ZSM-5 structure [12], are caused by insensitive internal tetrahedron asymmetric stretching vibrations and bending vibrations. The band at around 800 cm^−1^ can be attributed to both structure sensitive external tetrahedron and structure insensitive internal tetrahedron symmetric stretching vibrations. Meanwhile, the characteristic band of the double five rings structure of MFI-type zeolites could be observed at 620 cm^−1^ from all the samples, which can be used to estimate the crystalline degree of the samples. Although the band of the subnanocrystals at 620 cm^−1^ is weak, it may be proofed that the subnanocrystals could have the primary structure of MFI-type zeolites [9].

### The esterification conversion

**Fig. 3:**
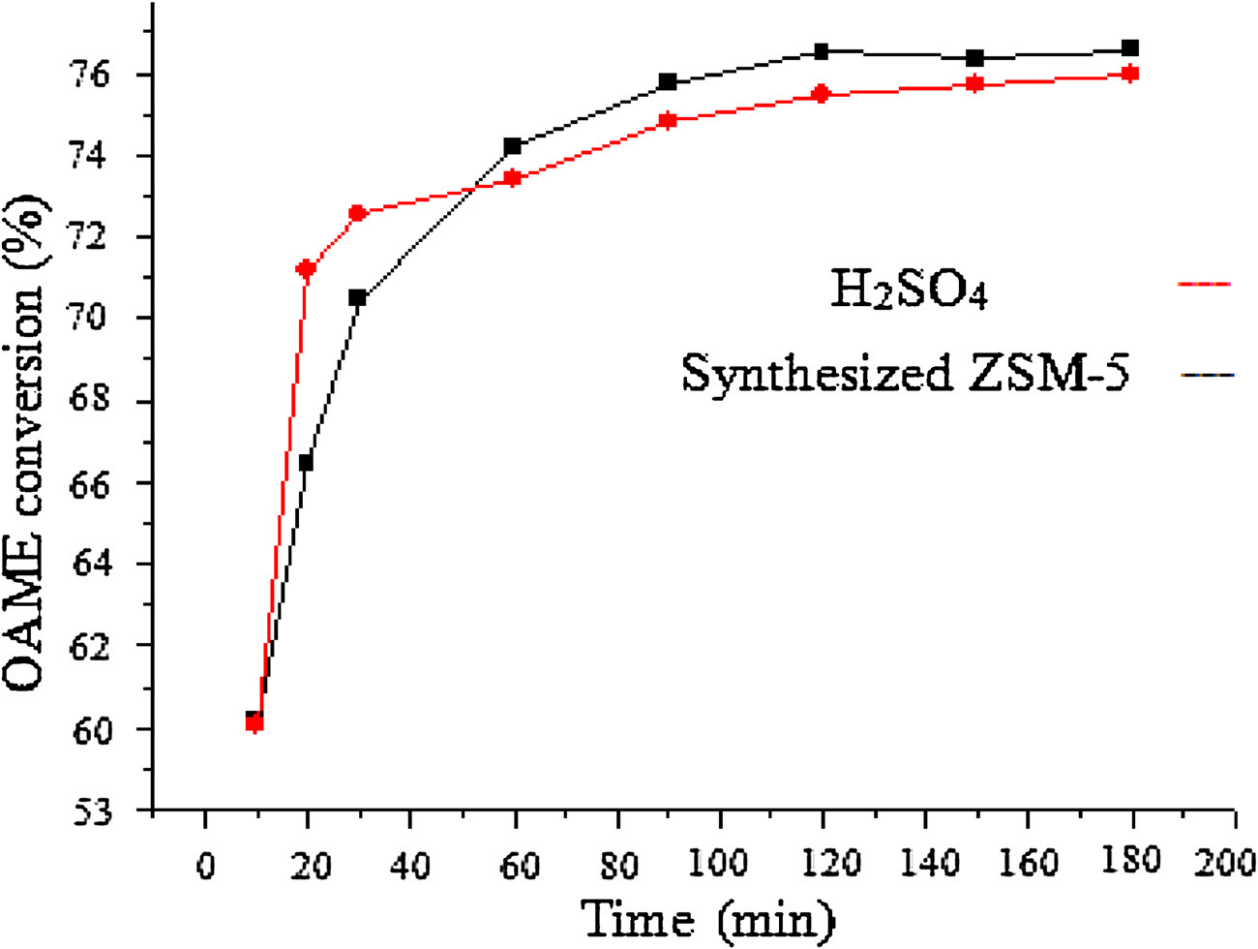
OAME conversions with synthesized ZSM-5 and H_2_SO_4_ catalysts.

The conversion of oleic free fatty acid into Oleic acid methyl ester was determined by measuring the acid value at the end of the reactions. To compare the catalytic behavior of the two catalysts in the reaction using the same molar amount of sulfuric acid (homogeneous catalysis) and synthetized ZSM-5 (heterogeneous catalysis) was performed (Fig. 3).

Using H_2_SO_4_ catalyst, excellent yields of OAME, 60%, 71% and 72.5% were obtained at 10, 20 and 30 min respectively. However, in the same conditions the OAMEs were obtained with lower yields (60–70%) using ZSM-5 catalysts. The higher esterification rate of ZSM-5 can be attributed to its stronger acidity compared to H_2_SO_4_, especially after 50 min of reaction. At 60 min, using the same catalyst… the conversion ratio was 74% while, under the same conditions, reached 50% in previous work [13].

The esterification rate is believed to be determined by the acidity strength and pore size of the catalyst [8]. Generally, the acidic strength is determined by the zeolite crystal structure. In addition, zeolites have a high hydrophobicity. Hydrophilic zeolites absorb a considerable amount of water in esterification. Water content limits the maximum conversion that can be achieved. Either, it is better likely the reason that Lewis acid sites removed and Bronsted acid sites, responsible for catalytic activity in esterification reactions, formed [[14], [15]].

## Conclusion

The synthesis of ZSM-5 can be realized with a simple hydrothermal method with modifications result a subnanocrystals which can catalysis an esterification of a free fatty acid with short chain to produce biodiesel with a performance better to this observed in commercialize Zeolite. The catalyst was shown to be efficient and promising for the esterification of oleic acid with methanol. ZSM-5 can be than an excellent alternative to sulfuric acid which caused corrosion and equipments damage.

## Funding

This research did not receive any specific grant from funding agencies in the public, commercial, or not-for-profit sectors.

## Conflict of interest

No conflict of interests including financial, personal or other relationships with other people or organizations.
